# Comparative assessment of bone mineral density levels in type 2 diabetic subjects with or without chronic periodontitis: A cross-sectional study

**DOI:** 10.34172/japid.2021.008

**Published:** 2021-06-09

**Authors:** Hira Ateeq, Afaf Zia, Qayyum Husain, Afshan Bey

**Affiliations:** ^1^Department of Biochemistry, Faculty of Life Science, Aligarh Muslim University, Aligarh, India; ^2^Dr Ziauddin Ahmad Dental College, Aligarh Muslim University, Aligarh, India

**Keywords:** Bone resorption, Dual-energy x-ray, Absorptiometry scan, Glycated hemoglobin A, Inflammation, Osteoporosis

## Abstract

**Background:**

This cross-sectional study investigated the bone mineral density (BMD) in type 2 diabetes mellitus (T2DM) subjects with or without chronic periodontitis (CP).

**Methods:**

A total of 120 subjects aged 35‒55, divided equally into four groups: i) T2DM with CP, ii) T2DM without CP, iii) CP alone, and iv) healthy patients, were included in this study. Clinical parameters like plaque index (PI), gingival index (GI), and probing pocket depth (PPD) were recorded. All the participants were evaluated for blood sugar levels using glycated hemoglobin (HbA1c) and BMD by Hologic dual-energy x-ray absorptiometry (DEXA) scan. The association of BMD with clinical periodontal parameters and HbA1c in all groups was investigated using linear correlation analysis (r).

**Results:**

The mean value of BMD (0.9020±0.0952 g/cm2) was lower in subjects with both T2DM and CP compared to T2DM and CP alone. BMD was weakly correlated with all the clinical periodontal parameters; a positive correlation was observed between BMD and GI in the T2DM and CP group (r=0.405, *P*=0.026) and the CP group (r=0.324, *P*=0.081). A weak positive correlation was observed in BMD and HbA1c in the T2DM group (r=0.261, *P*=0.13), T2DM and CP group (r=0.007, *P*=0.970), with a negative correlation to HbA1c in the CP group (r= -0.134, *P*=0.479).

**Conclusion:**

Diabetes mellitus impacts clinical periodontal status and bone mass, and the effect is accentuated when chronic periodontitis is present. Based on the present study, BMD is associated with T2DM and CP, but a weak correlation was observed between BMD and HbA1c and clinical periodontal parameters.

## Introduction

Diabetes mellitus (DM) is a type of metabolic disorder characterized by a hyperglycemic state due to defects in insulin secretion, insulin activity, or both.^1^ Type 1 diabetes mellitus (T1DM) is marked by the destruction of beta (β) cells, while type 2 diabetes mellitus (T2DM) is associated with insulin resistance coupled with insufficient production of insulin.^2^ Hyperglycemic condition is followed by altered levels of circulating proteins such as collagens or lipids, which undergo non-enzymatic glycation and oxidation, resulting in advanced glycation end products (AGEs), upregulated activation of the pro-inflammatory transcription factor, nuclear factor-kappa B (NF-kB), and pro-inflammatory cytokines.^3^ Enhanced superoxide production by mitochondria and elevated levels of matrix metalloproteinases (MMPs) such as MMP-2 and 9 play an essential role in the pathogenesis of diabetes mellitus and its vascular complications.^4,5^ It is predicted that by 2025, around 380 million adults worldwide will be affected by diabetes, with a prevalence of 6.4%, which is a major global health concern.^6^

Chronic periodontitis (CP) is a sub-gingival infection mainly caused by gram-negative bacteria.^7,8^ There is an abnormal host response against the pathogens, and in its severe form, it is associated with bone loss and the loss of soft tissue attached to the tooth.^9,10^ Host-derived mediators such as prostaglandin (PG) E-2 along with pro-inflammatory cytokines such as interleukin (IL)-1, IL-6, and tumor necrosis factor (TNF)-α cause periodontal tissue breakdown, which is accentuated in the presence of diabetes.^11^ Risk factors associated with chronic periodontitis include cardiovascular diseases, poor glycemic control in patients with T2DM, complicated pregnancy, and osteoporosis.^12,13^

Among the plausible biological mechanisms that link diabetes and periodontitis is enhanced inflammation; thus, in diabetes mellitus subjects, there is increased production of advanced glycation end products (AGEs), which are accumulated in periodontal tissues.^14^ These AGEs interact with receptor activators of AGE (RAGE) on cells, such as macrophages, and stimulate the production of MMPs, adhesion molecules, and cytokines such as TNF-α, IL-1, 17, 6, 18, and other pro-inflammatory mediators.^15^ On the other hand, the systemic inflammatory response in periodontitis is characterized by dysregulated secretion of host-derived mediators of inflammation and tissue breakdown, further enhancing the hyperlipidemic diabetic state. Therefore, it is likely that upregulated inflammation arises from each condition adversely affects the other.^16^ All these factors result in local tissue damage, increased breakdown of the periodontal connective tissues, and resorption of alveolar bone, leading to the exacerbation of periodontitis.^17^

Bone remodeling is a complex process affected by various local and systemic factors.^18^ Bone formation is affected due to reduced expression of transcription factors that regulate osteoblast differentiation.^19^ BMD determined by bone turnover is one of the most predictive factors of osteoporotic fracture.^20^ Several mechanisms have been proposed to explain decreased bone mass in diabetes mellitus and periodontitis, such as disordered insulin secretion, increased levels of inflammatory mediators, and altered vitamin D and calcium levels.^13,21^ Accumulated AGEs in diabetes mellitus and periodontitis reduce osteoblast activity by the AGE-RAGE pathway and enhance osteoclastogenic activity by upregulating RANKL mRNA and impaired matrix mineralization.^22,23^ Activation of local immune and inflammatory responses results in increased secretion of cytokines such as interleukin-1 beta (IL-1β), TNF-α, and IL-6, increased oxidative stress, and disruption of the receptor activator of NF-κB ligand/osteoprotegerin (RANKL/OPG) axis to favor bone resorption, resulting in reduced bone mineral density.^24^ The existing literature documents that diabetes mellitus is linked with an increased risk of bone fracture and impaired healing.^25^ Prolonged inflammation in diabetes mellitus and periodontitis inhibits osteoblast differentiation and promotes osteoclastogenesis by producing lytic enzymes that negatively modulate bone homeostasis, increase bone resorption, and limit normal bone repair processes.^26^ Extension of periodontal inflammation to adjacent bone and ligament reduces BMD causing alveolar bone resorption.^27^ Reduced BMD in periodontitis can be linked to altered bone homeostasis due to systemic inflammation, reduced collagen synthesis, or hormone deficiencies such as estrogen or parathyroid hormone.^20^ Due to decreased insulin secretion, bone formation slows down in T1DM, while in T2DM, bone resorption becomes faster, resulting in decreased BMD and impaired mineralization and bone microarchitecture.^25^

Diabetic subjects have a higher prevalence of periodontitis as it is an established complication of diabetes mellitus. Similarly, periodontitis influences metabolic control and diabetic complications.^28^ The extent of the relationship of BMD with T2DM and chronic periodontitis alone has been studied widely, but its mechanism is still unclear. However, no direct association of BMD in diabetic patients with and without chronic periodontitis has been reported. Therefore, our study aimed to assess and compare BMD levels in T2DM subjects with or without chronic periodontitis to clarify the effect of systemic conditions on bone health. Correlation between BMD, glycated hemoglobin (HbA1c), and clinical parameters in T2DM subjects with or without chronic periodontitis was also elucidated.

## Methods

### Study design and population

For this cross-sectional study, 120 consecutive patients attending the clinics of the Department of Periodontics were evaluated based on eligibility for participation. Patients of both sexes aged 36‒55 were included. The study was approved by the Institutional Ethics Committee (D.NO.576/FM) and conformed to the STROBES guidelines for an observational study. All the patients were individually informed about the study, and informed consent forms were signed. The study was carried out following the ethical standards laid down in the 1975 Declaration of Helsinki as revised in 2008.

Diagnosis of T2DM was made according to the American Diabetes Association (ADA) criteria defining diabetes as HbA1c level ≥7%, and diagnosis of chronic periodontitis conformed to the classification by the American Academy Of Periodontology (2018). The patients were divided into four groups (n=30): group 1, systemically and periodontally healthy (N) (13 males, 17 females, 36‒51 years of age), group 2, patients with type 2 diabetes mellitus only (T2DM) (16 males, 14 females, 38‒51 years of age), group 3, patients with type 2 diabetes mellitus along with chronic periodontitis (T2DM+CP) (14 males, 16 females, 39‒54 years of age), group 4, patients with chronic periodontitis only (CP) (13 males, 17 females, 37‒52 years of age).

### Exclusion criteria

Pregnancy, lactating, early menopause, hormone replacement therapy, history of drug intake that affects BMD (thiazide diuretics, statins, anticoagulants, antiepileptics), diseases influencing bone metabolism (hypo/hyperthyroidism, Cushing syndrome, primary hyperparathyroidism, renal failure, liver disease, inflammatory bowel disease, malabsorption), alcohol consumption, osteoporotic fracture history, scoliosis, history of periodontal treatment within the last six months, surgical periodontal therapy, and antibiotic, anti-inflammatory, immunosuppressive or cytotoxic drug intake within the last three months. The demographic, clinical, and laboratory data were obtained from all the selected patients.

### Measurements of clinical parameters, BMD, HbA1c

Two suitably qualified examiners conducted periodontal examinations. All 32 teeth were examined. Probing pocket depth (PPD), plaque index (PI), gingival index (GI), and clinical attachment level (CAL) were measured at six sites per tooth (mesiobuccal, buccal, distobuccal, mesiolingual, lingual, and distolingual) using a UNC-15 probe. The CAL was calculated by measuring the distance of the PPD from the cementoenamel junction, adding the gingival recession, and subtracting gingival hyperplasia. BMD was performed using the non-invasive Hologic dual-energy x-ray absorptiometry (DEXA) method at Rajeev Gandhi Centre and expressed in g/cm^2^. Serum HbA1c levels were measured for all the selected subjects.

### Statistical analysis

Statistical analyses were carried out using MINITAB 19. All the data were expressed as means ± SD on a subject basis. The Kolmogorov-Smirnov test assessed the normality of all the groups. One-way ANOVA followed by post hoc Tukey tests determined the differences between the groups for parametric variables and Kruskal-Wallis test for non-parametric variables. Pearson’s correlation coefficient was used to determine the correlation between BMD and HbA1c and clinical periodontal parameters. P < 0.05 were considered statistically significant.

## Results

Table 1 shows demographic data, periodontal clinical parameters, HbA1c, and BMD status of the study groups. The mean BMD level (0.9020±0.0952) was lower in the T2DM+CP group than the T2DM and CP groups (0.9517±0.0760 and 0.9562±0.0810, respectively). All the periodontal clinical parameters exhibited significantly higher mean values in the T2DM+CP group than the CP and T2DM groups, suggesting faster periodontitis in T2DM patients. The mean HbA1c level (7.6167±0.4395) was markedly higher in the T2DM+CP group, supporting the evidence of a two-way relationship between T2DM and CP.

**Table 1:** Descriptive statistics of all clinical periodontal and diabetic parameters

**Factor (study groups)**		**T2DM** **(n=30)**	**T2DM+CP** **(n=30)**	**CP** **(n=30)**	**N** **(n=30)**
Demographic findings	Age (years)	45.433±4.400	44.677±3.871	45.300±4.170	44.00±3.76
	Gender (%)	M=53% F=47%	M=47% F=53%	M=43% F=57%	M=43% F=57%
Clinical parameters	Plaque Index (mm)	0.9900±0.2074	2.5733±0.2420	2.2767±0.2825	0.80±0.23
	Gingival Index	0.8600±0.2387	2.0533±0.2177	1.4800±0.2369	0.60±0.07
	Probing Pocket Depth (mm)	2.0100±0.4751	4.353±0.671	2.9933±0.3982	1.25±0.30
	Clinical Attachment Level (mm)	1.1267±0.4354	5.360±0.727	3.2733±0.3362	0.89±0.20
Investigations	HbA1c (%)	7.0400±0.3900	7.6167±0.4395	5.2100±0.3325	4.60±0.33
	BMD (gm/cm^2^)	0.9517±0.0760	0.9020±0.0952	0.9562±0.0810	-

Data were expressed as means ± SD. It can be seen that the mean BMD value is lower in the T2DM+CP group than in other study groups. Similarly, periodontal parameters are significantly higher in the T2DM+CP group. T2DM: Type 2 diabetes mellitus, CP: chronic periodontitis, N: control group, HbA1c: glycated hemoglobin, BMD: bone mineral density, gender expressed in %, M: male, F: female. Clinical parameters were expressed in mm.

Kolmogorov-Smirnov normality test was performed to discriminate between the effects of gender, age, HbA1c level, periodontal parameters, and BMD. All the periodontal parameters and age and gender were normal (parametric), with a P≤0.05 except for BMD (*P* > 0.05). The difference between the groups was analyzed using one-way ANOVA, followed by Tukey tests for multiple comparisons for normal parameters of PI, GI, PPD, CAL, HbA1c, and Kruskal Wallis test for the non-normal parameter, i.e., BMD, in all the study groups (Table 2).

**Table 2:** Difference between the groups for parametric and non-parametric parameters

**Parameter**	***P value*** *
PI	0.0000
GI	0.0000
PPD	0.0000
CAL	0.0000
HbA1c	0.0000
AGE	0.5030
GENDER	0.8920
BMD	0.014

*P < 0.05 was considered significant. All *P value*s are from one-way ANOVA, except for that of BMD, which is from Kruskal-Wallis Test. PI: plaque index, GI: gingival index, PPD: probing pocket depth, CAL: clinical attachment level, HbA1c: glycated hemoglobin, BMD: bone mineral density

Periodontal parameters, such as PI, GI, PPD, CAL, and HbA1c, were highly significant *P* = 0.000, suggesting diabetes as a risk factor in chronic periodontitis patients or indicating chronic periodontitis as a risk factor in T2DM patients. However, age and gender were not significant, suggesting that age and gender were less likely to affect disease progression or BMD levels. BMD was significant by Kruskal-Wallis test (*P* = 0.014), thus rejecting the hypothesis and suggesting that BMD has a relationship with periodontal disease and diabetes progression. Pearson’s correlation was performed to determine the extent of correlation.

### Correlation between BMD and other parameters

**Figure 1:**
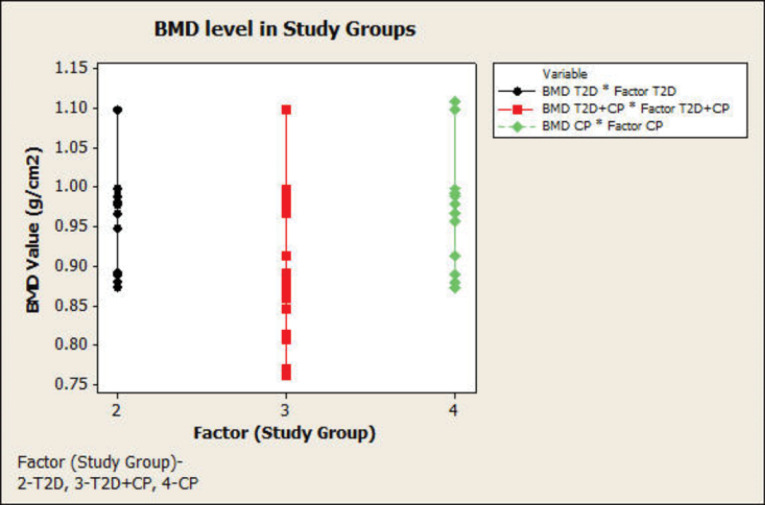
Correlation between bone mineral density (BMD) and Factor (Study Group). The scatterplot shows a lower BMD value in subjects with type 2 diabetes mellitus and chronic periodontitis (T2DM+CP) than other study groups. Factor 2. Study group patients with type 2 diabetes mellitus only (T2DM); Factor 3. Study group patients with type 2 diabetes mellitus and chronic periodontitis (T2DM+CP); Factor 4. Study group patients with chronic periodontitis only (CP).

**Figure 2:**
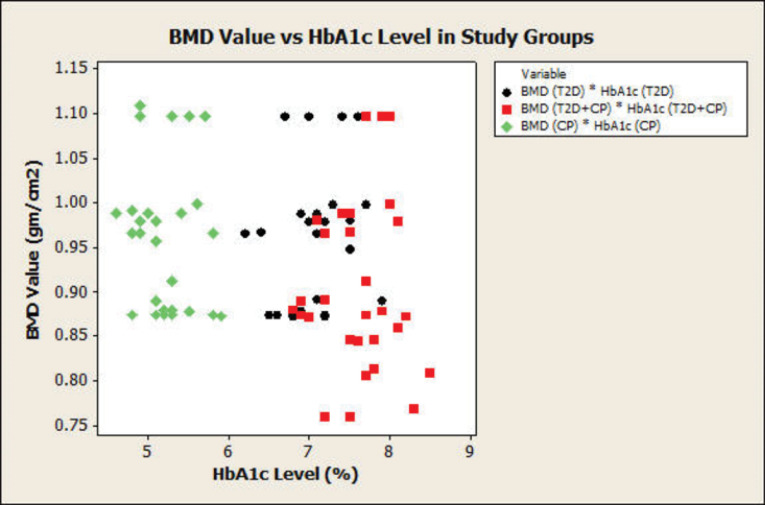
Correlation between bone mineral density (BMD) and glycated hemoglobin (HbA1c) in all the groups by scatterplot. As observed, with increasing levels of HbA1c, BMD value decreases, suggesting decreased BMD in diabetic subjects in all the study groups. Study group 2. Patients with type 2 diabetes mellitus only (T2DM); study group 3. Patients with type 2 diabetes mellitus and chronic periodontitis (T2DM+CP); study group 4. Patients with chronic periodontitis only (CP).

Table 3 shows the correlation of BMD with all the clinical periodontal parameters and HbA1c; however, not a very strong relationship could be observed. A weak negative correlation was observed in PI, GI, and BMD in T2DM patients, but there was a positive correlation in other groups. A strong positive correlation was seen between BMD and GI in T2D+CP and CP groups. BMD was weakly correlated with PD and CAL in other groups; however, there was a strong positive correlation between BMD and CAL in T2DM+CP. There were significant differences (*P* < 0.001) in clinical periodontal parameters in all the groups. There was a weak negative correlation between the HbA1c and BMD in the CP group. The BMD was negatively correlated with clinical periodontal parameters CAL and PPD. BMD was positively correlated with HbA1c in T2DM and weakly with T2DM+CP, with a weak negative correlation with CP. BMD was very weakly correlated with age in all the groups. Correlation analysis by a scatterplot showed lower BMD in T2D+CP compared to T2DM and CP groups (Figure 1). As observed by the scatterplot, BMD value decreased with increasing HbA1c levels, reaching a minimum in the T2DM+CP group, followed by T2DM and CP groups (Figure 2).

**Table 3:** Correlation (*r*) analysis between BMD and all the diabetic and periodontal parameters

**Correlation Parameters**	**T2DM**		**T2DM+CP**		**CP**	
	*r*-value	*P* value	*r*-value	*P* value	*r*-value	*P* value
BMD vs PI	-0.259	0.167	0.262	0.162	0.048	0.802
BMD vs GI	-0.123	0.517	0.405	0.026	0.324	0.081
BMD vs PPD	0.014	0.940	-0.141	0.458	0.091	0.633
BMD vs CAL	0.016	0.935	0.355	0.054	-0.047	0.807
BMD vs HbA1c	0.261	0.163	0.007	0.970	-0.134	0.479
BMD vs AGE	0.153	0.420	-0.317	0.087	0.076	0.691

*P<0.05 indicated a significant correlation. As can be seen, BMD shows a weak correlation between diabetes and clinical periodontal parameters.

## Discussion

This cross-sectional study was designed to evaluate BMD using Hologic dual-energy x-ray absorptiometry (DEXA) in males and females with T2DM with or without chronic periodontitis, who had no osteopenia or osteoporosis. DEXA scan is an accepted standard method for the measurement of bone mineral density clinically. It measures spine density versus peripheral density, unlike other non-invasive methods, such as photodensitometry, radiography, single-photon absorptiometry, and dual photon absorptiometry. Higher resolution, shorter scan time, and longer source life are some of its advantages. It uses x-rays produced by an x-ray cathode (low level) to estimate bone mineral content expressed in g/cm^2^.^29^ To the best of our knowledge, this is the first study that reports BMD in T2DM subjects with or without chronic periodontitis. The findings suggested a significant difference between diabetic subjects with or without periodontitis regarding BMD. Diabetic subjects with higher HbA1C levels exhibited more significant periodontal destruction, supporting pre-existing studies documenting a direct relationship between glucose and periodontitis progression.^30^ This study also supports existing literature that local mechanisms potentially contribute to decreasing the BMD of systemically healthy chronic periodontitis patients. As documented, cytokines, such as IL-1β and TNF-α, are potent stimulators of bone resorption in periodontitis and diabetes.^31^ HbA1c can be regarded as a positive factor for reduced BMD as higher glucose levels in blood generate an increased concentration of advanced glycation end products (AGEs), which possibly interact with the bone to reduce bone strength, resulting in osteoporosis in patients with diabetes mediated by apoptosis of osteoblasts, contributing to the defective bone formation and also hypercalciuria apart from microvascular complications of diabetes, which might lead to reduced blood flow to the bone, contributing to bone loss and fragility.^32^

Contradictory to our results, Oei et al^33^ reported a high bone mineral density in T2DM subjects, irrespective of poor glycemic control. In another study, the association between BMD and non-insulin-dependent diabetes mellitus (NIDDM) was assessed using DEXA; however, a strong relationship could not be concluded. Nonetheless, a slight decrease in bone mass was reported.^29^ Ibrahim et al^34^ reported an insignificant correlation between T2DM and bone mineral density values in men and women in a retrospective study conducted between 2010 and 2013, disregarding osteoporosis as a complication of T2DM. Still, the mechanism of how diabetes affects BMD is not clear; however, metanalysis have reported decreased markers of bone formation osteocalcin, bone-specific alkaline phosphatase, collagen type 1 C propeptide, and increased markers of bone formation markers of bone resorption deoxypyridinoline, C terminal cross-linked telopeptide, and N-terminal cross-linked telopeptide of type-I collagen in diabetic subjects, suggesting disturbed bone remodeling.^35^

Several studies have addressed the association between systemic BMD and periodontitis. Yoshihara et al^10^ reported a significant relationship between general BMD and periodontal disease in older adults. Some authors have reported an increase in BMD, others have reported a decrease, and a few have reported unaltered BMD status.^36,37^ A definite conclusion cannot be drawn regarding the extent of the relationship, whether controlling one factor will improve the other.

The present study had some limitations. The cause of the association between low BMD and T2DM and CP could not be manifested. It can be emphasized that the differences arise due to cross-sectional study design, limited sample size, disease complications, controlling the confounding factors, BMD measurement technology, sites of BMD measurement, and the methods used to assess periodontal disease or diagnose diabetes. The duration of the disease and BMI can also be crucial compounding factors but were not considered in the present study. Body mass index (BMI) was not calculated; the duration of diabetes affliction was not recorded. Although our study showed a correlation between BMD and glycated hemoglobin and clinical periodontal parameters in T2DM and CP patients, a definite relationship could not still be established.

## Conclusion

To summarize, our findings suggest that low BMD is associated with T2DM and chronic periodontitis. The early diagnosis of reduced BMD will significantly impact periodontal tissues. It can be considered an indicator of the existing periodontal condition, which needs to be examined by a dentist. Similarly, patients with T2DM should be evaluated for the risk of osteoporosis to reduce the risk of bone loss and fracture risk. Further longitudinal studies that show the associations between mandibular and skeletal BMD and the severity of periodontal disease in diabetes subjects and include larger study populations are required. BMD in individuals with diabetes with or without chronic periodontitis should be studied prospectively to elucidate the mechanism by which hyperglycaemic state might affect bone mass. Finally, interventional studies to investigate the effect on BMD and blood sugar levels in diabetics with or without chronic periodontitis will provide a more comprehensive understanding of bone remodeling.

## Author’s contributions

HA and AZ contributed to conception and design, analysis, and interpretation. HA drafted the manuscript. AZ contributed to drafting. HA, AZ, QH, and AB contributed to data acquisition and analysis. All the authors critically revised the manuscript, gave final approval, and agreed to be accountable for all aspects of the work, ensuring integrity and accuracy.

## Availability of data

The data that support the findings of this study are available upon request from the corresponding author, QH. The data are not publicly available due to restrictions, i.e., they contain information that could compromise the privacy of research participants.

## Ethics approval

Ethics Committee approval number is D.NO.576/FM.

## Competing interests

The authors report no conflict of interest related to the study.

## References

[1] Roden M (2016). Diabetes mellitus – Definition, Classification und Diagnossis. Wien Klin Wochenschr.

[2] Kaul K, Tarr JM, Ahmad SI, Kohner EM, Chibber R. Introduction to Diabetes Mellitus. In: Ahmad S.I. (eds) Diabetes. Advances in Experimental Medicine and Biology, vol 771. Springer 2013; New York, NY. 10.1007/978-1-4614-5441-0_1 23393665

[3] Yerneni KK, Bai W, Khan BV, Medford RM, Natarajan R (1999). Hyperglycemia- induced activation of nuclear transcription factor kappaB in vascular smooth muscle cells. Diabetes.

[4] Bulboaca AE, Porfire AS, Tefas LR, Boarescu PM, Bolboaca SD, Stanescu IC (2019). Liposomal Curcumin is Better than Curcumin to Alleviate Complications in Experimental Diabetic Mellitus. Molecules.

[5] Mohammad G, Siddiquei MM (2012). Role of matrix metalloproteinase-2 and -9 in the development of diabetic retinopathy. J ocul biol dis inform.

[6] Wild S, Roglic G, Green A, Sicree R, King H (2004). Global prevalence of diabetes: estimates for the year 2000 and projections for 2030. Diabetes care.

[7] Agrali OB, Kuru BE (2015). Periodontal treatment in a generalized severe chronic periodontitis patient: A case report with 7-year follow-up. Eur J Dent.

[8] Bastos Jdo A, Andrade LC, Ferreira AP, Barroso Ede A, Daibert Pde C, Barreto PL, Vilela EM, Marcaccini AM, Colugnati FA, Bastos MG (2013). Serum levels of vitamin D and chronic periodontitis in patients with chronic kidney disease. J Bras Nefrol.

[9] Khajuria DK, Zahra SF, Razdan R (2018). Effect of locally administered novel biodegradable chitosan based risedronate/zinc-hydroxyapatite intra-pocket dental film on alveolar bone density in rat model of periodontitis. J Biomater Sci Polym Ed.

[10] Yoshihara A, Seida Y, Hanada N, Miyazaki H (2004). A longitudinal study of the relationship between periodontal disease and bone mineral density in community-dwelling older adults. J Clin Periodontol.

[11] Anirudh BA, Srinath T, Muddapur MV, Raghavendra D, Kulkarni Kulkarni (2018). Systemic Cytokines in Type 2 Diabetes Mellitus and Chronic Periodontitis. Curr Diabetes Rev.

[12] Vestergaard P (2007). Discrepancies in bone mineral density and fracture risk in patients with type 1 and type 2 diabetes--a meta-analysis. Osteoporos Int.

[13] Tezal M, Wactawski-Wende J, Grossi SG, Ho AW, Dunford R, Genco RJ (2000). The relationship between bone mineral density and periodontitis in postmenopausal women. J Periodontol.

[14] Preshaw P, Bissett S (2019). Periodontitis and diabetes. Br Dent J.

[15] Litwinoff E, Hurtado DPC, Ramasamy R, Schmidt AM (2015). Emerging Targets for Therapeutic Development in Diabetes and Its Complications: The RAGE Signaling Pathway. Clin Pharmacol Ther.

[16] Taylor JJ, Preshaw PM, Lalla E (2013). A review of the evidence for pathogenic mechanisms that may link periodontitis and diabetes. J Clin Periodontol.

[17] Kayal RA, Siqueira M, Alblowi J, McLean J, Krothapalli N, Faibish D (2010). TNF-alpha mediates diabetes-enhanced chondrocyte apoptosis during fracture healing and stimulates chondrocyte apoptosis through FOXO1. J Bone Miner Res.

[18] Singh A, Sharma RK, Siwach RC, Tewari S, Narula SC (2014). Association of bone mineral density with periodontal status in postmenopausal women. J Investig Clin Dent,.

[19] Okamura H, Yoshida K, Ochiai K, Haneji T (2011). Reduction of protein phosphatase 2A Cα enhances bone formation and osteoblast differentiation through the expression of bone-specific transcription factor Osterix. Bone.

[20] Gordon CM, Zemel BS, Wren TAL, Leonard MB, Bachrach LK, Rauch F (2017). The Determinants of Peak Bone Mass. J Pedia.

[21] Brownlee M (2005). The pathobiology of diabetic complications: A unifying mechanism. Diabetes.

[22] Dereka XE, Markopoulou CE, Fanourakis G, Tseleni-Balafouta S, Vrotsos IA (2010). RANKL and OPG mRNA level after non-surgical periodontal treatment. Inflammation.

[23] Alharbi MA, Zhang C, Lu C, Milovanova TN, Yi L, Ryu JD (2018). FOXO1 Deletion Reverses the Effect of Diabetic-Induced Impaired Fracture Healing. Diabetes.

[24] Madianos PN, Koromantzos PA (2018). An update of the evidence on the potential impact of periodontal therapy on diabetes outcomes. J Clin Periodontol.

[25] Norris R, Parker M (2011). Diabetes mellitus and hip fracture: A study of 5966 cases. Injury.

[26] Weinberg E, Maymon T, Moses O, Weinreb M (2014). Streptozotocin-induced diabetes in rats diminishes the size of the osteoprogenitor pool in bone marrow. Diabetes Res Clin Pract.

[27] Saglam M, Koseoglu S, Hatipoglu M, Esen HH, Koksal E (2015). Effect of sumac extract on serum oxidative status, RANKL/OPG system and alveolar bone loss in experimental periodontitis in rats. J Appl Oral Sci.

[28] Llambes F (2015). Relationship between diabetes and periodontal infection. World J Diabetes.

[29] Ho SK, Eun SJ, Myung HY, Seoung-Oh Y (2018). Bone mineral density assessment for research purpose using dual energy X-ray absorptiometry. Osteoporosis and Sarcopenia.

[30] Sadeghi R, Taleghani F, Mohammadi S, Zohri Z (2017). The effect of diabetes mellitus type I on periodontal and dental status. J Clin Diagnostic Res.

[31] Wu YY, Xiao E, Graves DT (2015). Diabetes mellitus related bone metabolism and periodontal disease. Int J Oral Sci.

[32] Asokan AG, Jaganathan J, Philip R, Soman RR, Sebastian ST, Pullishery F. Evaluation of bone mineral density among type 2 diabetes mellitus patients in South Karnataka. J Nat Sci Biol Med 2017; 8(1):94–8. https://www.jnsbm.org/text.asp?2017/8/1/94/198363. 10.4103/0976-9668.198363 PMC5320831 28250682

[33] Oei L, Zillikens MC, Dehghan A, Buitendijk GH, Castano-Betancourt MC, Estrada K (2013). High bone mineral density and fracture risk in type 2 diabetes as skeletal complications of inadequate glucose control: the Rotterdam Study. Diabetes care.

[34] Ibrahim AA, Hanan YA, Amnah A, Atheer A, Aisha A (2015). Bone Mineral Density (BMD) in patients with type 2 diabetes mellitus. Adv Environ Biol.

[35] Li KH, Liu YT, Yang YW, Lin YL, Hung ML, mLin IC (2018). A positive correlation between blood glucose level and bone mineral density in Taiwan. Arch Osteoporos.

[36] Xu S, Zhang G, Guo JF, Tan YH (2021). Associations between osteoporosis and risk of periodontitis: A pooled analysis of observational studies. Oral Dis.

[37] Qiu J, Li C, Dong Z, Wang J (2021). Is diabetes mellitus a risk factor for low bone density: a systematic review and meta-analysis. BMC Endocr Disord.

